# HYDROSAFE: A Hybrid Deterministic-Probabilistic Model for Synthetic Appliance Profiles Generation †

**DOI:** 10.3390/s24175619

**Published:** 2024-08-29

**Authors:** Abdelkareem Jaradat, Muhamed Alarbi, Anwar Haque, Hanan Lutfiyya

**Affiliations:** The Department of Computer Science, Western University, London, ON N6A 3K7, Canada; malarbi@uwo.ca (M.A.); ahaque32@uwo.ca (A.H.); hlutfiyy@uwo.ca (H.L.)

**Keywords:** appliance operation modes, demand response (DR), dynamic time warping (DTW), HEMSs, load profile simulation, SHEMSs

## Abstract

Realistic appliance power consumption data are essential for developing smart home energy management systems and the foundational algorithms that analyze such data. However, publicly available datasets are scarce and time-consuming to collect. To address this, we propose HYDROSAFE, a hybrid deterministic-probabilistic model designed to generate synthetic appliance power consumption profiles. HYDROSAFE employs the Median Difference Test (MDT) for profile characterization and the Density and Dynamic Time Warping based Spatial Clustering for appliance operation modes (DDTWSC) algorithm to cluster appliance usage according to the corresponding Appliance Operation Modes (AOMs). By integrating stochastic methods, such as white noise, switch-on surge, ripples, and edge position components, the model adds variability and realism to the generated profiles. Evaluation using a normalized DTW-distance matrix shows that HYDROSAFE achieves high fidelity, with an average DTW distance of ten samples at a 1Hz sampling frequency, demonstrating its effectiveness in producing synthetic datasets that closely mimic real-world data.

## 1. Introduction

This era of dependence on fossil fuels and concern about greenhouse gas emissions has recently increased interest in utilizing new solutions in the Smart Grid (SG) to decrease energy consumption [1]. The residential sector’s contribution to total energy usage has increased from 22% of the total energy consumption in the USA in 2009 [2] to approximately 27% in 2020 [3]. In Canada, the residential sector accounted for 28% of total energy usage in 2006, while it accounted for 32% in 2020. It is expected that residential use will continue to rise with the same pattern until 2050 [3]. Particularly, home appliances are responsible for a decent portion of energy usage; in the USA, home appliances consume approximately 30% of the total household consumption [4], while in Canada, home appliances account for 14% of total household consumption [5].

One of the common strategies that offers many integrated solutions to reduce electric usage in the residential sector is the utilization of Smart Home Energy Management Systems (SHEMSs) [6]. SHEMSs are multi-component systems that focus on energy monitoring, analysis, scheduling, storage [7,8] and feedback based on several inputs such as electricity tariffs, appliances power data collected by sensors, power consumption limit, tenants preferences, occupancy rates, and environmental data [9]. SHEMSs utilize different approaches to analyze these inputs such as Machine Learning (ML) [10] and Digital Signals Processing (DSP) methods which result in providing user feedback, appliances scheduling, user information systems [11,12]. All these outcomes focus on having users understand household usage better and promote energy sustainability [13] using different approaches such as Demand Response (DR) [14].

Within SHEMSs, to develop and validate the aforementioned analytical algorithms and methods, representative datasets are needed. Power Consumption Datasets (PCDs) [15] play a crucial role in this context. PCDs are datasets containing time-series data corresponding to samples of the instantaneous power consumption for electric loads. PCDs come into aggregated and disaggregated forms. The aggregated form is when more than one load or all the loads within the same residence are measured into a single time-series. The other form is the disaggregated PCDs when each load in the house is individually measured into a separate time-series [16]. Despite the recent efforts in collecting residential PCDs [15], public PCDs availability is still limited [17,18,19,20] due to the need to setup measurement devices within households and the long time it takes for data collection which may take years in some cases [15]. In many cases, the publicly available PCDs do not satisfy the necessity for PCDs that holds appliance-specific features to support validating data analysis algorithms [14]. To overcome this issue, researchers use Synthetic PCDs (SyPCDs) [17,21], which have the potential to extend PCDs and save the installation cost and measurement time [20]. SyPCDs are generated load profiles for household appliances based on either publicly available PCDs (deterministic) or on mathematical (probabilistic) models [22]. SyPCDs should be realistic in representing the original dataset, tunable, expandable, and unbiased. In this work, a Hybrid Deterministic-Probabilistic Model for Synthetic Appliance Profiles Generation (HYDROSAFE) is proposed. HYDROSAFE is a hybrid deterministic-stochastic model that is built to extend existing PCDs and aims to simulate household appliances’ usage profiles when activated with different Appliance Operation Modes (AOMs), which represent specific settings set to the appliance to meet the customers needs. At its core, the main objective of HYDROSAFE is to generate realistic appliance power consumption time series data based on a hybrid model. This model incorporates both deterministic methods that are built on top of a data analysis of publicly available PCDs, and probabilistic methods, which adds stochasticity to the model to maximize the realistic aspect of the generated data. The ultimate goal of HYDROSAFE is to generate Synthetic SUPs (SySUPs) in different AOMs for household appliances.

**Figure 1:**
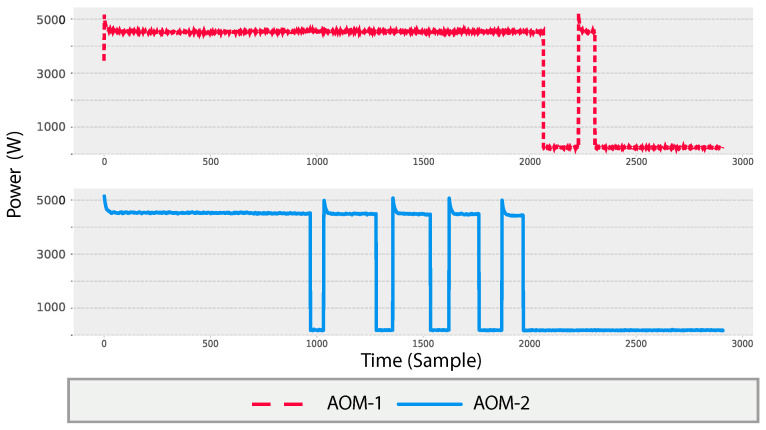
Two SUPs for a clothes dryer. Each SUP is activated with a different AOM.

**Figure 2:**
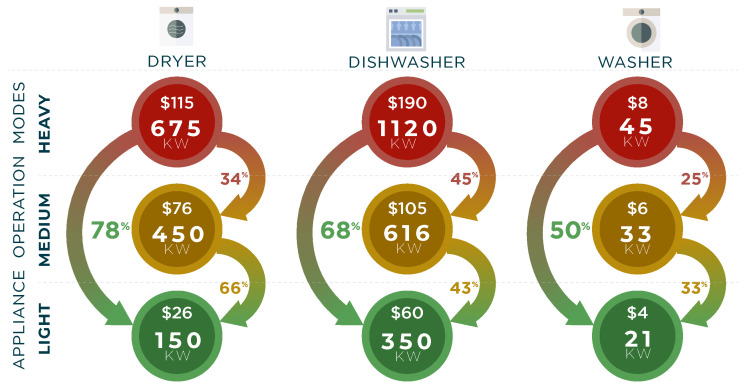
The annual power consumption [23] and corresponding costs for operating 3 appliances, and the potential savings by switching to the lighter operation modes.

In domestic settings, home appliances often operate in distinct modes tailored to specific user needs and circumstances. An Appliance Operation Mode (AOM) denotes a predefined configuration established by the appliance manufacturer to accommodate user preferences across varying scenarios. Each AOM is characterized by its duration of operation and the unique cycles and states through which the appliance transitions. For instance, consider a dishwasher equipped with three operation modes: a light setting for lightly soiled dishes, a medium setting for moderately soiled dishes, and a heavy setting for heavily soiled dishes. The consumption of electricity varies depending on the specific AOM activated for a given appliance. Figure 1 illustrates two instances of Synthetic Usage Profiles (SUPs) for a clothes dryer, each corresponding to the appliance being activated with different AOMs. Figure 2 shows the average annual power consumption with the associated cost for three appliances within the same household. The figure shows the potential saving percentages achieved in load reduction by switching the use of appliances from heavy to medium, medium to light, and heavy to light AOMs. For example, if a household switches from using heavy to medium modes in a dishwasher, 45% of the cost is cut, while if the shifting occurs from heavy towards a light mode, 68% of the consumption is reduced annually [23].

The rest of the paper is organized as follows: In Section 2, previous work in the literature is presented. In Section 3, the problem formulation is elaborated. Section 4 presents the architecture of HYDROSAFE. In Section 5, SUPs extraction and smoothing is discussed. Section 6 presents the formal characterization of SUPs. Section 7 discusses the operation modes clustering using DTW algorithm. In Section 8, the process of generating synthetic SUPs is presented. Section 9 presents the evaluation of HYDROSAFE. Finally, Section 10 concludes the paper and suggests future work.

## 2. Related Work

Many of the available methods [17,24,25] of simulating appliance usage profiles focus on the consumer’s behavioral patterns [26], and determine the power consumption based on the occupancy actions [27] or based upon a psychological model [20]. A popular simulator is CREST [28], which is based on a combination of active occupancy patterns and profiles of daily activity that describes the patterns of occupants activities. An extension model [29] is built on top of CREST integrates a new thermal-electrical model into the existing model.

A probabilistic-empirical residential electricity load model [19] is designed to generate 1 min intervals power use of appliances based on both measured and statistical data besides occupant activities such as cooking, watching TV, etc. A stochastic approach [30] is used in the generation of high-resolution multi-energy load profiles for residential loads in remote areas. A mathematical framework [31] is developed for simulating household appliances by re-synthesizing the current waveforms, harmonic currents and the phase shifting of the appliances. Similar work [21] uses GUI in Matlab Simulink to simulate household loads.

Generative Adversarial Networks (GANs) [32] are rapidly evolving in many disciplines, including synthetic PCDs generation. TraceGAN [33] and PowerGAN [34] by Harell et al., ProfileSR-GAN [35] by Song et al., mREAL-GAN [36] by Sanderson et al., RLPGen [37] by Liang et al., SGAN [38] by Gkoutroumpi et al., are examples of recent literature works that explores the realm of generating realistic appliance data using GANs. GANs are known to be data-hungry [39] and require large datasets to be trained [40]. Since available PCDs with labeled AOMs is still very small [14], HYDROSAFE does not focus on using GANs. Language models are also used to generate PCDs as an N-gram language model based approach is proposed in [41] by obtaining a string representations of electricity consumption time series data from a household appliance, and then creating a unigram and bigram for each appliance category.

Several synthetic datasets for the residential sector is available. Table 1 lists recent publicly available residential and commercial SyPCDs and their basic characteristics. The Automated Model Builder for Appliance Loads (AMBAL) [42] is a load simulation tool designed to extract appliance models from real datasets. These models are composed of sequences of parameterized signatures, and play a crucial role in simulating a realistic household environment through the use of a trace generator. This synthetic appliance trace generator enables the recombination of appliance models, thus facilitating the simulation of user activities in homes with customizable complexity. A similar approach is used in SynD dataset [43], but for a larger number of appliances. SmartSim [44] is a device-accurate home energy load generator. It utilizes device energy and device usage models to simulate a household loads using a sequence of Distribution learning, Event marking, and Trace Generation components. SmartSim leverages its modeling by build on the data from Smart* energy dataset [45]. Other datasets, such as SHED [46,47] contains data for commercial buildings.

**Research gap:** The literature extensively examines methods for simulating residential load profiles [24,53]. However, to the best of the authors’ knowledge, no prior research has explicitly focused on simulating household appliances within the context of Appliance Operation Modes (AOMs). This observation underscores a notable limitation in the existing literature, revealing a significant gap in understanding and addressing the dynamics of appliance behavior within diverse operational modes.

**Academic contribution:** In this work, HYDROSAFE fills this gap by presenting a novel open-source [54] hybrid deterministic-probabilistic approach. HYDROSAFE leverages both empirical data and sophisticated statistical models to generate appliance usage profiles encompassing multiple operation modes. This development is crucial, as it provides a more accurate representation of real-world appliance usage, thereby enhancing the effectiveness of energy management systems and algorithms. Furthermore, simulating AOMs offers an opportunity to improve methods for analyzing power consumption data with a focus on AOMs, enabling households to achieve more significant energy savings.

## 3. Problem Formulation

The purpose of HYDROSAFE is to generate a synthetic dataset of household appliance usage profiles. This section describes the definitions and formulation for the generation process.

It is assumed that a household, *h*, belongs to the household set, H, such that:

(1) $$\begin{array}{c} h\in H=\{h_{1},\ldots ,h_{i},\ldots ,h_{\left|H\right|}\}\\ 1\leq i\leq |H| \end{array}$$

where the household set, H, is of size, |H|. A household, *h*, runs an appliance, *a*, that belongs to the set of appliances, $A^{h}$, such that:

(2) $$\begin{array}{c} a\in A^{h}=\left\{a_{1},\ldots ,a_{i},\ldots ,a_{|A^{h}|}\right\}\\ 1\leq i\leq |A^{h}| \end{array}$$

where $|A^{h}|$ is the number of appliances in the household, *h*.

An operation mode, *p*, that belongs to the operation modes set, $P^{a}$, is defined as follows:

(3) $$\begin{array}{c} p\in P^{a}=\left\{p_{1},\ldots ,p_{i},\ldots ,p_{|P^{a}|}\right\}\\ 1\leq i\leq |P^{a}| \end{array}$$

where $|P^{a}|$ is the number of operation modes available for appliance, *a*. It is assumed that a complete run for an appliance, *a*, starts and ends in the same day. A day, *d*, is defined as:

(4) $$\begin{array}{c} d\in D=\{d_{1},\ldots ,d_{\left|D\right|}\}\\ 1\leq i\leq |D| \end{array}$$

where the set of days, D, is of size, |D|. A daily power consumption sequence, $\Omega_{a}^{d}$, represents the power consumption samples taken in a single day, *d*, for the appliance, *a*, such that:

(5) $$\begin{array}{c} \Omega_{a}^{d}={\left\{\omega_{n}\right\}}_{n=1}^{n_{*}}=\left\{\omega_{1},\omega_{2},\ldots ,\omega_{n},\ldots ,\omega_{n_{*}}\right\}\\ 1\leq n\leq n_{*} \end{array}$$

where $\omega_{n}$ is the $n^{th}$ instantaneous power sample value measured in (W), and $n_{*}$ represents the last sample index within *d*. When a sampling frequency, $f_{s}$, is used, the value of $n_{*}$ for a single day is defined as follows:

(6) $$n_{*}=t.f_{s}$$

where *t* is the time measured in seconds. For a single day, t=86,400 s.

A Single Use Profile (SUP) is used to formally model the power consumption of a preprogrammed appliance between the time it is turned on and the time it is switched off. A SUP represents the sequence (or time-series) of the power consumption values (measured in Watts) consumed by an appliance from the moment of turning it on to the moment of turning it off. A SUP, $\psi^{p}$, with length $|\psi^{p}|$, is defined by the sequence of samples that represent a subsequence of the daily consumption, $\Omega_{a}^{d}$, from the moment of turning the appliance on, $n^{s}$, to the moment that it is turned off, $n^{e}$. This is defined as:

(7) $$\begin{array}{c} \psi^{p}={\left\{\omega_{n}\right\}}_{n=n^{s}}^{n^{e}}=\left\{\omega_{n^{s}},\omega_{n^{s}+1},\ldots ,\omega_{n},\ldots ,\omega_{n^{e}-1},\omega_{n^{e}}\right\}\\ \psi^{p}\subseteq \Omega_{a}^{d}\;,\;n^{s}\leq n\leq n^{e}\leq n_{*} \end{array}$$

where:

(8) $$\begin{array}{c} |\psi^{p}|=n^{e}-n^{s}+1 \end{array}$$

and for all SUPs, $\psi^{p}\subseteq \Omega_{a}^{d}$, there is no overlapping between any two SUPs such that the intersection between these SUPs.

The set of SUPs, $\Psi_{a}^{d,p}$, of size $|\Psi_{a}^{d,p}|$, that corresponds to appliance *a*, and labeled by the AOM *p*, is defined as:

(9) $$\begin{array}{c} \Psi_{a}^{d,p}=\left\{\psi_{1}^{p},\ldots ,\psi_{j}^{p},\ldots ,\psi_{|\Psi_{a}^{d,p}|}^{p}\right\} \end{array}$$

where $\Psi_{a}^{d,p}$ contains all the SUPs that run using the same AOM, *p*. The set of all SUPs, $\Psi_{a}^{d}$, in *d* is defined as the union of all disjoint subsets $\Psi_{a}^{d,p}$ corresponding to every AOM $p\in P^{a}$. This is defined as:

(10) $$\begin{array}{c} \Psi_{a}^{d}=\overset{|P^{a}|}{\underset{i=1}{⋃}}\Psi_{a}^{d,p_{i}}=\left\{\psi_{1}^{p_{j}},\ldots ,\psi_{i}^{p_{k}},\ldots ,\psi_{Z_{a}^{d}}^{p_{l}}\right\}\\ \left\{p_{j},p_{k},p_{l},\ldots \right\}\in P^{a}\\ s.t,\;\overset{|P^{a}|}{\underset{i=1}{⋂}}\Psi_{a}^{d,p_{i}}=\phi \end{array}$$

where $\Psi_{a}^{d}$ is a set of size, $|\Psi_{a}^{d}|$, that represents the total size of all AOM subsets, such that:

(11) $$\begin{array}{c} |\Psi_{a}^{d}|=\sum_{p\in P^{a}}\left|\Psi_{a}^{d,p}\right| \end{array}$$

The main objective of HYDROSAFE is to generate a set of Synthetic SUPs (SySUPs) that can be used to validate the analytical methods to support Demand Response (DR) [14]. The set of SySUPs, ${\ddot{\Psi }}_{a}$, are generated by the HYDROSAFE modules, such that:
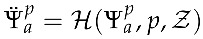
(12)
where H is the HYDROSAFE generator function, *p* is the selected AOM to generate SUPs in, $\Psi_{a}^{p}$ is the set of extracted SUPs from the dataset, and 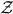 is the set of tuning parameters used in the generation process.

## 4. HYDROSAFE Architecture

**Figure 3:**
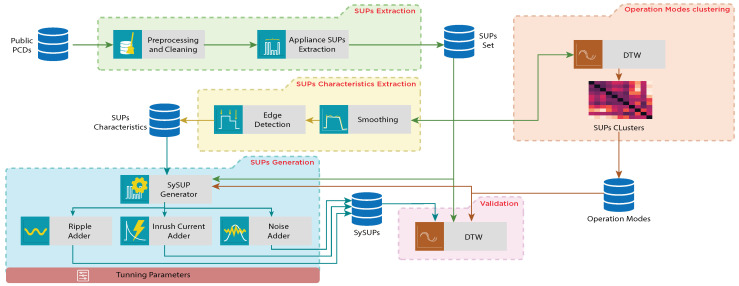
The architecture of HYDROSAFE.

The architecture of HYDROSAFE depicted in Figure 3 comprises five main components: First, the *SUPs Extraction* module processes a publicly available PCD [23] and extracts a set of Single Use Profiles (SUPs) for all appliances represented in the PCD. The *SUP Characteristics Extraction* module applies a series of processes on the set of extracted SUPs to identify the characteristics of SUPs in a formal model. The *Operation Modes Clustering* component applies the Dynamic Time Warping (DTW) algorithm [55] among all SUPs so that these SUPs are grouped into clusters, each of which contains SUPs that are associated with the same AOM. The *SUP Generation* component is responsible for synthesizing the Synthetic SUPs (SySUPs) using the set of extracted SUPs, the SUPs characteristics, and the AOMs. This module is composed of multiple submodules, each of which accounts for a particular part in the formation of SySUPs. Finally, the *Validation* module is used to evaluate the similarity of the resulting SySUPs compared to the extracted SUPs. Figure 3 illustrates the architecture of HYDROSAFE.

## 5. SUPs Extraction and Smoothing

This section discusses the process of extracting SUPs from the time series data [23] to be used in the synthesis of SySUPs.

### 5.1. SUPs Extraction

All SUPs are extracted from the Rainforest Automation Energy Dataset (RAE) by Makonin et al. [23] on a daily basis per appliance. This dataset is chosen because it is extensively used in numerous works within the literature, making it a well-established and validated source of data for research on appliance power consumption. The extraction process is based on XCorrelation [56], which is based on this previous work [14]. For any appliance, the daily consumption sequence, $\Omega_{a}^{d}$, contains zero or more non-overlapping SUPs, such that:

(13) $$\begin{array}{c} \psi_{i}^{p_{\alpha }}={\left\{\omega_{n}\right\}}_{n=n^{s_{i}}}^{n^{e_{i}}}\;,\;\psi_{j}^{p_{\beta }}={\left\{\omega_{n}\right\}}_{n=n^{s_{j}}}^{n^{e_{j}}}\\ i<j\;\forall \;\left\{i,j\right\}\in \{1,2,\ldots ,|\Psi_{a}^{d}|\}\\ 1\leq n^{s_{i}}<n^{e_{i}}<n^{s_{j}}<n^{e_{j}}\leq n_{*} \end{array}$$

where the first SUP, $\psi_{i}^{p_{\alpha }}$, runs with the operation mode, $p_{\alpha }$, and starts at $n=n^{s_{i}}$ and ends at $n=n^{e_{i}}$. The other SUP, $\psi_{i}^{p_{\beta }}$, runs with the operation mode, $p_{\beta }$, and starts at $n=n^{s_{j}}$ and ends at $n=n^{e_{j}}$. The activation time of each SUP is before the switch off time as $n^{s_{i}}<n^{e_{i}}$, $n^{s_{j}}<n^{e_{j}}$, and both SUPs do not overlap throughout the day, such that $n^{e_{i}}<n^{s_{j}}$. The power consumption samples that belong to the time intervals between SUPs are defined as follows:

(14) $$\begin{array}{c} \omega_{n}\leq \tau^{\varepsilon }\;\forall \;n\in \left\{n^{e_{i}}+1,n^{e_{i}}+2,\ldots ,n^{s_{j}}-1\right\} \end{array}$$

where $\tau^{\varepsilon }$ is a threshold that represents the stand-by power which corresponds to the appliance state when the appliance is switched off or switched to a stand-by state. In this state, the appliance consumption is closest to zero as the consumption is very minimal, which is caused by low-rated appliance components such as Light Emitting Diodes (LED lights).

### 5.2. SUPs Smoothing

Typically, any signal can be decomposed into multiple components. Signal components can be high or low in frequency. In this context, a high frequency component with relatively low amplitude is considered noise, and needs to be reduced [57]. The moving median smoother is used to reduce the high frequency component [58]. To reduce the high frequency component within SUPs, a transformation function is applied on the SUP sequence, ψ, of length, |ψ|, to generate the smoothed SUP, $\hat{\psi }$, of length, $|\hat{\psi }|$. The moving median smoother, M, is selected. This transformation is performed as follows:

(15) $$\begin{array}{c} \hat{\psi }\left(n\right)=M\left(\psi \left(k\right)\right) \end{array}$$

where *W* is the sliding window size that is used by M. By using a sliding window transformation, the transformed sequence length is shortened by a factor of the window size, *W*, where two sequences of length $\left\lfloor \frac{W}{2}\right\rfloor$ are padded to $\hat{\psi }$ to compensate the decrease in length caused by the transformation. A leading sequence is prepended before $\hat{\psi }$, where each prepended item is equal to the first sample of $\hat{\psi }$, i.e., $\hat{\psi }\left(1\right)$. A lagging sequence is appended after $\hat{\psi }$, where each appended item is the last sample of $\hat{\psi }$, i.e., $\hat{\psi }\left(|\hat{\psi }|\right)$. This is shown as follows:

(16) $$\begin{array}{c} {\hat{\psi }}_{\mathrm{after}}=\underset{\left\lfloor \frac{W}{2}\right\rfloor }{\underset{︸}{\left\{\hat{\psi }\left(1\right),\ldots ,\hat{\psi }\left(1\right)\right\}}}\;\cup \;{\hat{\psi }}_{\mathrm{before}}\;\cup \;\underset{\left\lfloor \frac{W}{2}\right\rfloor }{\underset{︸}{\left\{\hat{\psi }\left(|\hat{\psi }|\right),\ldots ,\hat{\psi }\left(|\hat{\psi }|\right)\right\}}} \end{array}$$

where ${\hat{\psi }}_{\mathrm{before}}$ is the smoothed SUP before padding, and ${\hat{\psi }}_{\mathrm{after}}$ is the smoothed SUP after padding. After this concatenation, the length of the transformed SUP, $|\hat{\psi }|$, will be equal to the length of the SUP, |ψ|.

**Figure 4:**
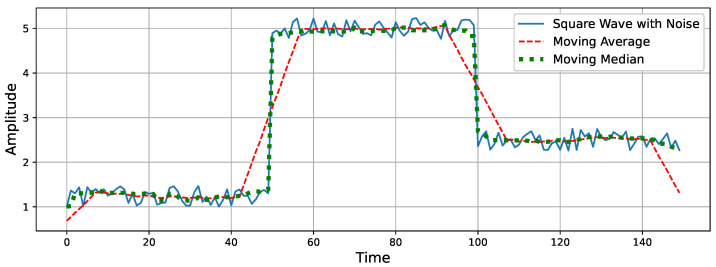
A square wave with uniform noise, moving average, and moving median.

The choice of the moving median smoother in this context stems from its advantageous property of edge preservation. When processing SUPs characterized by square waveforms with minor fluctuations, the precise location of edges holds significance in delineating SUP characteristics. Unlike alternative smoothers like the moving mean, the moving median exhibits superior edge preservation capabilities. Specifically, the moving mean introduces distortions to edges, resulting in the transformation of vertical edges into skewed counterparts. This distortion introduces an inherent uncertainty, thereby compromising the precision of SUP characteristics delineation. As such, the utilization of the moving median smoother ensures enhanced accuracy in defining SUP characteristics by mitigating edge distortion effects. In Figure 4, a square wave with added noise is illustrated. The smoothed wave obtained using the moving average exhibits a skewness in the edges. Conversely, the smoothed wave obtained using the moving median effectively preserves the edges.

**Figure 5:**
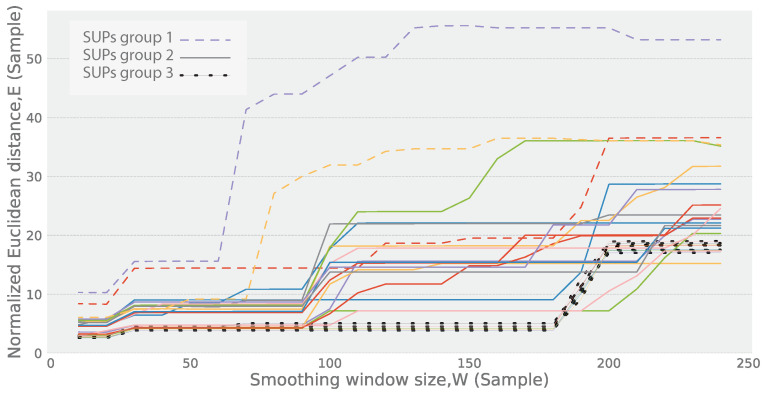
The Euclidean distance between trimmed vs. smoothed multiple SUP for a dryer using moving median with variation in the window size.

Selecting the smoothing window size, *W*, impacts the degree of similarity between ψ and $\hat{\psi }$. Higher values of *W* produce wider windows and, therefore, significantly reduce the high frequency noise, while they may cause deformation in the major shape (the low frequency component) of $\hat{\psi }$, which results in a higher distance. On the other hand, lower values of *W* produce narrower windows and, therefore, the high frequency noise may not be sufficiently eliminated, which may also result in a higher distance. To measure this impact, according to Tan et al. [59], the Minkowski distance, particularly the L2 norm of the Minkowski distance, is utilized to measure this impact, namely the Normalized Euclidean Distance [59]. This is defined as follows:

(17) $$\begin{array}{c} E\left(\psi ,\hat{\psi }\right)=\frac{1}{\left|\psi \right|}\sqrt{\sum_{k=1}^{\left|\psi \right|}{\left|\psi \left(k\right)-\hat{\psi }\left(k\right)\right|}^{2}} \end{array}$$

where the function, *E*, measures the normalized pairwise distance between the points, ψ(k) and the corresponding point, $\hat{\psi }\left(k\right)$, assuming that $\left|\psi |=\right|\hat{\psi }|$. Figure 5 illustrates the effect of varying the window size, *W*, on the distance, $E(\psi ,\hat{\psi })$. The window size *W* spans from 5 to 250 samples, and the distance *E* is depicted for 25 SUPs. A higher value on the plot indicates a greater difference in $E(\psi ,\hat{\psi })$, suggesting that some of the significant topographies of ψ have been smoothed out. Conversely, as the plot flattens, it signifies that the smoothing function has retained all major topographies defining the shape of ψ while eliminating the noise component. These patterns shown in the figure highlight how the SUPs respond differently based on the characteristics of the states being removed and the corresponding window size. The plot presents three distinct groups of SUPs, each exhibiting a staircase-like response to the distance, *E*. This staircase pattern emerges due to the progressive removal of short states from the SUPs as the window size increases. Group 1 shows an early step up in the plot, which corresponds to the removal of shorter states from the SUP. As the window size increases slightly, these short states are excluded, resulting in a noticeable increase in the plot. Group 2 and Group 3 demonstrate steps that occur at larger window sizes. In these cases, the removal of states with wider durations causes the step in the plot to appear later, as it requires a larger window size for these wider states to be excluded. The initial disparity in $E(\psi ,\hat{\psi })$ stems from the elimination of spikes at the beginning of each state (referred to as the inrush current [60]), which typically contributes to a brief period of high power consumption.

## 6. Extraction of SUPs Features

In major appliances, a SUP consists of a sequence of activations and deactivations of the internal components of the appliance. For example, in the clothes dryer, the heating element and the spinning motor switch on and off during the operation cycle. During the activation of such internal elements, the appliance is considered to be in a particular state.

A state corresponds to the power values recorded during the activation of these components over specific time interval. This process of switching the appliance’s components on and off results into a sudden changes in the power consumption, which show as a common pattern in the corresponding SUP as a sequence of square-like wave with abrupt changes (edges) between the states. The exact sample when the abrupt change occurs is defined as Exact Edge. When detecting these exact edges, the detection mechanism firstly specifies a Thick Edge, which is an interval that surrounds the exact edge with high likelihood indication of the existence of the exact edge.

The number of states, their distributions, durations, and power levels are all characteristics that determine the features of SUPs of a specific AOM compared to other AOMs within the process of generating SySUPs. The following subsections describe the steps to determine the characteristics of SUPs in terms of the states that form these SUPs.

### 6.1. Estimation of State Edges

An indicator vector, *I*, is used to determine the bounds of each state in the SUP, $\hat{\psi }$. The Median Difference Test (MDT) [61] is used to calculate the values of the indicator vector, *I*. MDT utilizes a moving window with length, $W^{I}$, that slides over the subsequences of $\hat{\psi }$. The MDT estimates the presence of an exact edge within $\hat{\psi }$ by dividing the moving widow into two equal length partitions. The median, M, is evaluated for each partition along with the standard deviation, σ, of the entire window. The indicator vector is defined as the following:

(18) $$\begin{array}{c} I^{\hat{\psi }}\left(n\right)=\sqrt{\sigma \left(\hat{\psi }\left(n^{i}\right)\right)\left|M{\left(\hat{\psi }\left(n^{j}\right)\right)}^{2}-M{\left(\hat{\psi }\left(n^{q}\right)\right)}^{2}\right|}\\ \forall \;n^{i}\in k\;,\;\forall \;n^{j}\in k^{l}\;,\;\forall \;n^{q}\in k^{r}\\ k^{l}=\left\{n-\frac{W^{I}}{2},n-\frac{W^{I}}{2}+1,\ldots ,n\right\}\;,\;k^{r}=\left\{n+1,\ldots ,n+\frac{W^{I}}{2}-1,n+\frac{W^{I}}{2}\right\}\\ k=k^{l}\cup k^{r}\\ \forall \;n,\;1\leq n\leq |\hat{\psi }| \end{array}$$

where $I^{\hat{\psi }}$ is the indicator vector corresponding to the SUP, $\hat{\psi }$. $M(\hat{\psi }\left(n^{j}\right))$ is the median of the SUP samples corresponding to the left partition of the window as of $n^{j}\in k^{l}$, where $k^{l}$ is the list of sample indexes of the left window. $M(\hat{\psi }\left(n^{q}\right))$ is the median of the SUP samples correspond to the right partition of the window as of $n^{q}\in k^{r}$, where $n^{q}$ is the list of indexes of the right window. $\sigma (\hat{\psi }\left(n^{i}\right))$ is the standard deviation of the SUP samples of the entire window as of $n^{i}\in k$.

The evaluated value of $I^{\hat{\psi }}\left(n\right)$ is proportional to the likelihood of having an exact edge in $\hat{\psi }$ at sample index *n*. When the window falls within a rising edge, the value of the left partition median, $M(\hat{\psi }\left(k^{q}\right))$, is lower that the value of the right partition median, $M(\hat{\psi }\left(k^{q}\right))$. The difference between the two medians will be relatively high. Additionally, since there is a large difference between the left and right partitions values, the indicator value, $I^{\hat{\psi }}$, increases by a factor of the standard deviation of the entire window, $\sigma (\hat{\psi }\left(k^{i}\right))$. The leading and lagging gaps caused by the moving window is padded using the same technique used in Equation (16).

**Figure 6:**
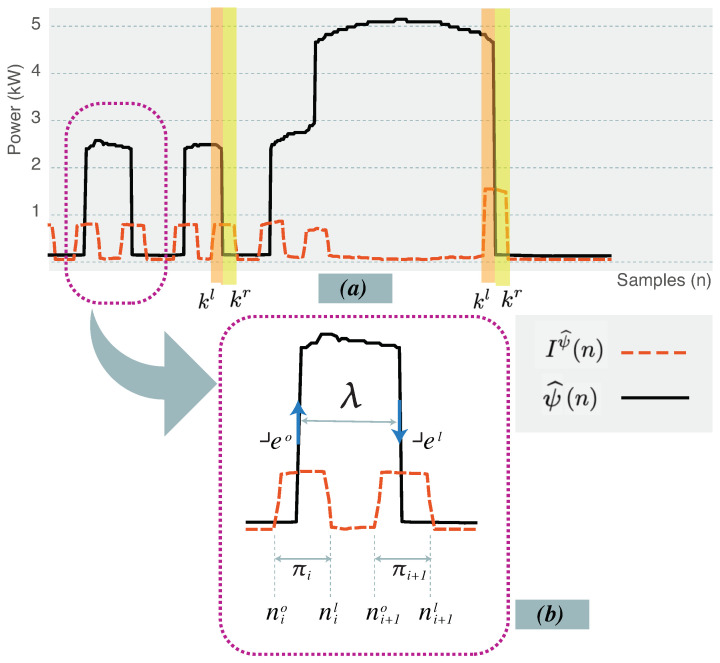
(**a**) The smoothed SUP sequence $\hat{\psi }\left(n\right)$ with the indicator vector $I^{\hat{\psi }}\left(n\right)$. (**b**) A zoom-in to a state showing its upper and lower bounds, the upper and lower bounds of thick edges, the exact edges.

The indicator vector, $I^{\hat{\psi }}$, is depicted in Figure 6. The plot of $I^{\hat{\psi }}$ shows two states: A steady low amplitude values that indicate a steady behavior in $\hat{\psi }$ as the change in the value of $\hat{\psi }$ is relatively small. The other state is a list of spikes that indicate a higher likelihood that an abrupt change in the value of $\hat{\psi }$ has occurred. The starting and ending sample indexes of each of the spikes bound the exact edge in $\hat{\psi }$. These two bounds form a thick edge, which is a pair of sample indexes that indicates the presence of a rising of falling exact edges in $\hat{\psi }$. The sequence of thick edges, $\Pi^{\hat{\psi }}$, of size $|\Pi^{\hat{\psi }}|$, that is identified by $I^{\hat{\psi }}$ in $\hat{\psi }$, is defined as follows:

(19) $$\Pi^{\hat{\psi }}={\left\{\pi_{i}\right\}}_{i=1}^{|\Pi^{\hat{\psi }}|}\;,\;\left|\Pi^{\hat{\psi }}\right|\leq \frac{|\hat{\psi }|}{2}$$

where $\pi_{i}$ is the $i^{th}$ thick edge that is defined as the following pair of sample indexes:

(20) $$\pi_{i}=\left(n_{i}^{o},n_{i}^{\iota }\right)\;,\;1\leq n_{i}^{o}\leq n_{i}^{\iota }\leq \left|\hat{\psi }\right|$$

where $\pi_{i}$ defines two boundaries, $n_{i}^{o}$ as the lower bound, while the upper bound is $n_{i}^{\iota }$. The sequence of thick edges, $\Pi^{\hat{\psi }}$ is obtained by applying a threshold, $\tau^{I}$, so that a thick edge, π, is defined as the following:

(21) $$\begin{array}{c} I^{\hat{\psi }}\left(n\right)\leq \tau^{I}\;,\;\forall \;n\in \left\{n_{j}^{\iota }+1,n_{j}^{\iota }+2,\ldots ,n_{k}^{o}\right\}\\ \pi_{j}=\left(n_{j}^{o},n_{j}^{\iota }\right)\;,\;\pi_{k}=\left(n_{k}^{o},n_{k}^{\iota }\right)\\ 1\leq n_{j}^{o}\leq n_{j}^{\iota }<n_{k}^{o}\leq n_{k}^{\iota }\leq \left|\hat{\psi }\right| \end{array}$$

where $\pi_{j}$, $\pi_{k}$ are two consecutive thick edges.

### 6.2. Determining SUP States

A SUP encompasses a succession of states representing the power consumption behavior of internal electrical components within an appliance. Each state within the sequence corresponds to the power values recorded during the activation of these components over specific time intervals. These sequences tend to exhibit a relatively stable pattern with slight variations, reflecting the consistent power consumption behavior of the internal components.

The sequence of states, Λ, that is associated with $\hat{\psi }$ is defined as follows:

(22) $$\begin{array}{c} \Lambda^{\hat{\psi }}=\left\{\lambda_{1},\ldots ,\lambda_{i},\ldots ,\lambda_{\left|\Lambda \right|}\right\}\\ \lambda_{i}=\left(e^{o},e^{\iota },\omega^{\lambda }\right)\;,\;e^{o}<e^{\iota }\\ \lambda_{1}=\left(1,1,\omega_{1}^{\lambda }\right)\;,\;\lambda_{\left|\Lambda \right|}=\left(\right|\hat{\psi }\left|,\right|\hat{\psi }\left|,\omega_{|\hat{\psi }|}^{\lambda }\right) \end{array}$$

where the state, $\lambda_{i}$, is represented by a tuple with three elements: the left exact edge, $e^{o}$, the right exact edge, $e^{\iota }$, and the power value, $\omega^{\lambda }$. The values of the exact edges are determined through the process of Edge Thinning. In this process, the exact edge, *e*, is evaluated from the corresponding thick edge, π. One method for edge thinning is argmax method where the value of *e* equals the sample index that produces the maximum indicator vector value, $I^{\hat{\psi }}$. This is defined as:

(23) $$\begin{array}{c} e_{i}^{o}=argmax\left(I^{\hat{\psi }}\left(n\right)\right)\;,\;\forall n\in \pi_{i}\\ e_{i}^{\iota }=argmax\left(I^{\hat{\psi }}\left(n\right)\right)\;,\;\forall n\in \pi_{i+1}\\ i\in \{1,3,5,\ldots \} \end{array}$$

where the left exact edge, $e_{i}^{o}$, is selected from a thick edge, $\pi_{i}$, i.e., $e_{i}^{o}\in \pi_{i}$ and the right exact edge, $e_{i}^{\iota }$, is selected from a thick edge, $\pi_{i+1}$, i.e., $e_{i}^{\iota }\in \pi_{i+1}$.

The other method of edge thinning is the mid-point method. The center point of the thick, $\pi_{i}$, is selected as the exact edge. This is shown as follows:

(24) $$\begin{array}{c} e_{i}^{o}=\frac{n_{i}^{o}+n_{i}^{\iota }}{2}\;,\;e_{i}^{\iota }=\frac{n_{i+1}^{o}+n_{i+1}^{\iota }}{2}\\ i\in \{1,3,5,\ldots \} \end{array}$$

As both of the left and right exact edges of the state, $\lambda_{i}$ are determined, the power value of the state, $\omega_{i}^{\lambda }$, is evaluated as the median, M, of the power values in $\hat{\psi }$ corresponding to each sample index in the state, $\lambda_{i}$. This is defined as the following:

(25) $$\begin{array}{c} \omega_{i}^{\lambda }=M\left({\left\{\omega_{k}\right\}}_{k=e_{i}^{o}}^{e_{i}^{\iota }}\right) \end{array}$$

where $\omega_{k}$, is the power values of $\hat{\psi }$ at the sample index, *k*.

The sequence of states, Λ, that represents $\hat{\psi }$ is defined as follows:

(26) $$\begin{array}{c} \Lambda^{\hat{\psi }}=\left\{\lambda_{1},\ldots ,\lambda_{i},\ldots ,\lambda_{\left|\Lambda \right|}\right\}\\ \lambda_{i}=\left(e_{i}^{o},e_{i}^{\iota },\omega^{\lambda }\right) \end{array}$$

such that Λ contains all the features that distinguish one SUP from another. These features are used for SUP classification and clustering [62].

## 7. SUP Clustering

Typically, disaggregated PCDs comprise a set of SUPs per appliance where each SUP represents an activation with a particular AOM. This section discusses how to use DTW to group SUPs that belong to the same AOM in clusters [63]. The DTW distance measure is used in HYDROSAFE as a measure of similarity among SUPs. This similarity is used to create clusters of appliance SUPs where the members of each cluster share the same operation mode. The DTW distance is calculated between any two temporal sequences, *Q* and *P*, such that $Q={\left\{q_{i}\right\}}_{i=1}^{n}$ and $P={\left\{p_{j}\right\}}_{j=1}^{m}$. DTW maps the elements of *Q* and *P* to minimize the distance between *Q* and *P* based on a warping path [55], $W\in W={\left\{w_{k}\right\}}_{k=1}^{K}$ over all possible paths, W. This is defined as follows:

(27) $$\begin{array}{c} DTW\left(Q,P\right)=\min_{\begin{array}{c} W\\ W\in W \end{array}}\left\{\sum_{k=1}^{K}ℵ\left(w_{k}\right)\right\}\\ \max(m,n)\leq K<m+n-1 \end{array}$$

where $w_{k}=\left(i,j\right)$ is the pair of indices, and $ℵ(w_{k})$ is the distance element that belongs to the warping path *W* that aligns the elements of both *Q* and *P* such that the distance between them is minimized. The minimization is obtained through iteratively minimizing the distance of the current element and the adjacent elements within the distance matrix, *ℵ*, such that:

(28) $$\begin{array}{c} ℵ\left(i,j\right)=d_{e}\left(q_{i},p_{j}\right)+\min\left\{ℵ\left(i-1,j-1\right),ℵ\left(i-1,j\right),ℵ\left(i,j-1\right)\right\} \end{array}$$

where $d_{e}\left(q_{i},p_{j}\right)$ is the Euclidean distance between $q_{i}$ and $p_{j}$. The optimal warping path is found by tracing *ℵ* backward, choosing the adjacent points with the lowest distance [55].

Generally, SUPs that belong to a specific operation mode have a smaller DTW distance among each other, and hence, higher similarity. On the other hand, pairwise DTW distance which is the distance between two SUPs that belong to different operation modes have higher distance and thus less similarity. To ensure the uniformity in the distances, the normalized pairwise DTW distance, $\bar{\delta }$, between two SUPs, $\psi^{p_{i}}$ and $\psi^{p_{j}}$, is defined as the following:

(29) $$\begin{array}{c} \bar{\delta }\left(\psi^{p_{i}},\psi^{p_{j}}\right)=\frac{DTW\left(\psi^{p_{i}},\psi^{p_{j}}\right)}{|\psi^{p_{i}}\left|+\right|\psi^{p_{j}}|}\\ p_{i},p_{j}\in P^{a}\;,\;\psi^{p_{i}}\in \Psi_{a}^{d,p_{i}}\;,\;\psi^{p_{j}}\in \Psi_{a}^{d,p_{j}} \end{array}$$

The distance matrix, Δ, is a $\left(|\Psi_{a}^{d}|\times |\Psi_{a}^{d}|\right)$ square matrix that contains the normalized pairwise distances, $\bar{\delta }$, for all SUPs $\psi^{p}\in \Psi_{a}^{d}$. The distance matrix is defined as:

(30) $$\begin{array}{c} \Delta =\\ \left[\begin{array}{ccc} \begin{array}{c} \left[\begin{array}{c} \Delta_{i,j}=\bar{\delta }\left(\psi_{i}^{p_{0}},\psi_{j}^{p_{0}}\right)\\ i,j\in \{1,\ldots ,|\Psi_{a}^{d,p_{0}}|\} \end{array}\right] \end{array} & & \\ & \left[\begin{array}{c} \ddots \end{array}\right] & \\ & & \begin{array}{c} \left[\begin{array}{c} \Delta_{i,j}=\bar{\delta }\left(\psi_{i}^{p_{k}},\psi_{j}^{p_{k}}\right)\\ i,j\in \{|\Psi_{a}^{d,p_{k-1}}|,\ldots ,|\Psi_{a}^{d,p_{k}}|-|\Psi_{a}^{d,p_{k-1}}|\} \end{array}\right] \end{array} \end{array}\right] \end{array}$$

where the distance matrix, Δ, is a symmetric square matrix, i.e., $\Delta_{i,j}=\Delta_{j,i}$ since each of its elements is compared to all other elements in Δ. The element $\Delta_{i,j}$ represents the distance between $\psi_{i}^{p_{k}}$ and $\psi_{j}^{p_{l}}$, such that:

(31) $$\begin{array}{c} \Delta_{i,j}=\bar{\delta }\left(\psi_{i}^{p_{k}},\psi_{j}^{p_{l}}\right)\\ \forall \left\{\psi_{i}^{p_{k}},\psi_{j}^{p_{l}}\right\}\in \Psi_{a}^{d} \end{array}$$

Since Δ is a symmetric square matrix, the upper triangle in Δ contains the same values in the lower triangle. The diagonal values of Δ are defined as:

(32) $$\begin{array}{c} \Delta_{i,j}=0\;,\;\forall \;i=j \end{array}$$

which refers to the zero distance between a SUP and itself.

The SUPs clustering is based on the Density And Dynamic Time Warping Based Spatial Clustering For Appliance Operation Modes (DDTWSC) [63]. DDTWSC is a clustering algorithm for SUPs based on the DTW distance. DDTWSC utilizes two hyperparameters: ϵ,μ [64] where ϵ is the Eps-neighborhood hyperparameter that determines the maximum radius in which all SUPs within that radius will be considered for further steps. μ represents the minimum number of adjacent elements located in the given region surrounded by ϵ. The clustering of the SUPs is modeled as follows:

(33) $$\begin{array}{c} \left\{{\left\{\Psi_{a}^{p}\right\}}^{p\in P},\Psi_{a}^{o}\right\}=DDTWCS\left(\Psi_{a},\mu ,\epsilon \right) \end{array}$$

such that, by the end of the algorithm, the set of all SUPs, $\Psi_{a}$ is clustered into $|P_{a}|$ clusters. Each cluster, $\Psi_{a}^{p}$, contains all SUPs that belongs to the operation mode, *p*, while the outlier SUPs set is $\Psi_{a}^{o}$.

Based on the calculated matrix, $\Delta_{i,j}$, a subset of directly density-reachable SUPs, $\Psi_{a}^{*}$, is selected from $\Psi_{a}$ that contains all SUPs, $\psi_{j}$, with a distance, $\delta (\psi_{i},\psi_{j})$, less than a predetermined threshold, ϵ, such that:

(34) $$\begin{array}{c} \Psi_{a}^{*}={\left\{\psi_{j}\in \Psi_{a}|\bar{\delta }\left(\psi_{i},\psi_{j}\right)<\epsilon \right\}}_{i=j=1}^{|\Psi_{a}|,j\neq i} \end{array}$$

The next step in the clustering process is when the next hyperparameter, μ, is used. Three different subsets are formed upon applying μ and ϵ on $\Psi_{a}^{*}$, namely the core SUPs set, $\Psi_{a}^{c}$, the border SUPs set, $\Psi_{a}^{b}$, and the outlier SUPs set, $\Psi_{a}^{o}$. These subsets are defined as follows corresponding to the value of μ:

(35) $$\begin{array}{c} \left\{\begin{array}{cc} \Psi_{a}^{o}⇐\Psi_{a}^{o}\cup \Psi_{a}^{*}, & |\Psi_{a}^{*}|=0\\ \Psi_{a}^{b}⇐\Psi_{a}^{b}\cup \Psi_{a}^{*}, & 0\leq |\Psi_{a}^{*}|<\mu \\ \Psi_{a}^{c}⇐\Psi_{a}^{c}\cup \Psi_{a}^{*}, & |\Psi_{a}^{*}|\geq \mu \end{array}\right. \end{array}$$

where $\Psi_{a}^{o}$ contains the SUPs that are considered outliers to any cluster. $\Psi_{a}^{c}$ contains the core SUPs which incrementally joins other core SUPs in further iterations until it forms a cluster, $\Psi_{a}^{P}$.$\Psi_{a}^{b}$ contains the border SUPs which may join other core sets in further iterations or remain as-is until the end of the algorithm. By The end of the algorithm, the set of all SUPs, $\Psi_{a}$, is clustered into $|P_{a}|$ clusters. Each cluster, $\Psi_{a}^{p}$, contains all SUPs that belongs to the operation mode, p.

Assuming that SUPs are grouped in Δ by the operation mode, each row in Δ contains a set of values that are more similar to each other in the same operation mode, such that:

(36) $$\begin{array}{c} \bar{\delta }\left(\psi^{p_{i}},\psi^{p_{j}}\right)\approx \bar{\delta }\left(\psi^{p_{i}},\psi^{p_{k}}\right),\;p_{i}=p_{j}=p_{k} \end{array}$$

while it also contains a set of values that are relatively larger. That correspond to distances with SUPs among different operation modes. This is defined as:

(37) $$\begin{array}{c} \bar{\delta }\left(\psi^{p_{i}},\psi^{p_{j}}\right)<<\bar{\delta }\left(\psi^{p_{i}},\psi^{p_{k}}\right),\;p_{i}=p_{j}\neq p_{k} \end{array}$$

**Figure 7:**
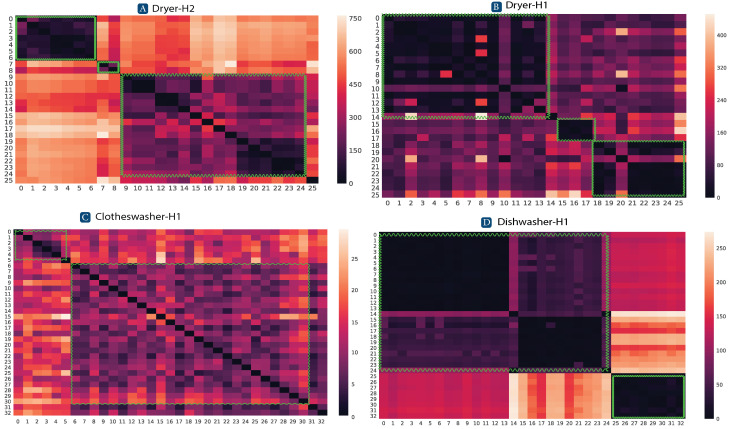
The distance matrix, Δ, for 3 appliances in 2 houses [23]. (**A**) A dryer in house-2 with 3 AOMs. (**B**) A dryer in house-1 with 3 AOMs. (**C**) A clothes washer in house-1 with 2 AOMs. (**D**) A dishwasher in house-1 with 2 AOMs.

A heat map is used to visualize the operation modes based on the pairwise distance among them. Figure 7 shows the distance matrix, Δ, for three appliances. The heatmap shows a gradient that refers to the degree of similarity between two corresponding SUPs. Darker color implies higher similarity while lighter color implies less similarity. For example, in Figure 7A, the dryer shows three different operation modes, as it shows three darker areas surrounded by squiggly lines. According to Equation (37), if we consider SUP-0, it is observed that the distance, $\bar{\delta }$, between SUP-0 and SUPs 1 to 6 is very minimal. This means that high similarity exists among this cluster of SUPs, i.e., these SUPs share the same operation mode. On the other hand, according to Equation (37), the same SUP-0 has higher distance with SUPs 7 to 8 and 9 to 25, since these SUPs belong to different operation modes. A summary of the statistics of the pairwise DTW distances for the RAE dataset [23] are listed in Table 2 and Table 3.

## 8. Generating Synthetic SUPs

A set of Synthetic SUPs (SySUPs), ${\ddot{\Psi }}_{a}^{p}$, is defined as:

(38) $$\begin{array}{c} {\ddot{\Psi }}_{a}^{p}={\left\{{\ddot{\psi }}_{i}\right\}}_{i=1}^{|{\ddot{\Psi }}_{a}^{p}|} \end{array}$$

where ${\ddot{\psi }}_{i}$ is a SySUP for the appliance, *a*, with operation mode, *p*. The value of $|{\ddot{\Psi }}_{a}^{p}|$ is selected to be a large number (typically $|{\ddot{\Psi }}_{a}^{p}|>1000$) for evaluation purposes.

A base SySUP, $\ddot{\psi }\in {\ddot{\Psi }}_{a}^{p}$, represents a SySUP without adding the effect of any additional tuning parameters. The base SySUP, $\ddot{\psi }$, is defined as follows:

(39) $$\begin{array}{c} \ddot{\psi }={\left\{{\left\{\omega_{j}^{\lambda }\right\}}_{j=1}^{e_{\lambda }^{\iota }-e_{\lambda }^{o}}\right\}}^{\lambda \in \Lambda^{{\hat{\psi }}^{p}}} \end{array}$$

where $\omega^{\lambda }$ is the power value of the state, $\lambda \in \Lambda^{{\hat{\psi }}^{p}}$. $\Lambda^{{\hat{\psi }}^{p}}$, is a randomly selected SUP with operation mode, *p*, and $e_{\lambda }^{\iota }-e_{\lambda }^{o}$ is the length of the state, λ. The following subsections discuss the probabilistic components that is added to the base SySUP.

The base SySUP, $\ddot{\psi }$, forms the foundation of our synthetic dataset. It consists of sequences of power values, $\omega_{j}^{\lambda }$, which represent the power consumption in different states λ of an appliance *a* operating in mode *p*. The set $\Lambda^{{\hat{\psi }}^{p}}$ includes all possible states λ that an appliance can be in during the operation mode *p*.

### 8.1. The White Noise Component

Real-world PCDSs often includes noise due to various factors like sensor inaccuracies or environmental influences causing the states of a SUP not to be completely flat. To simulate this, and to better capturing the variability and stochastic nature of real-world appliance usage patterns, a random noise is added to the power values $\omega_{j}^{\lambda }$. This noise can be modeled as a small random perturbation, typically drawn from a normal distribution centered around zero. The noise coefficient, ξ, is defined as follows:

(40) $$\begin{array}{c} \xi_{j}=N\left(\mu^{\xi },\sigma^{\xi }\right) \end{array}$$

where $\xi_{j}$ is the added noise to the $j^{th}$ sample in the base SySUP, $\ddot{\psi }$. This noise is selected based on a normal distribution function, N, with a mean of $\mu^{\xi }$ and standard deviation $\sigma^{\xi }$. The set of SySUPs with the added noise, Ψ¨apξ, is defined as:

(41) Ψ¨apξ=ψ¨iξi=1|Ψ¨apξ|

where the resulting SySUP with the added noise, ψ¨ξ, is defined as:

(42) ψ¨ξ=ωjλ+ξjj=1eλι−eλoλ∈Λψ^p,ψ¨ξ∈Ψ¨apξ

### 8.2. The Switch-On Surge Component

The Switch-On Surge (SOS), or Inrush Current, is the maximum instantaneous input current consumed by electrical transformers within an electrical device when first switched on [60]. This phenomenon typically occurs within the first few samples of high-power states when a major component within the appliance is triggered. The SOS component exhibits distinct characteristics that are important to understand for accurate modeling and analysis. SOS is characterized by a sharp initial peak at the beginning of a state, which then decays in amplitude over time. The High Initial Peak occurs when an electrical device, particularly one with inductive loads such as motors or transformers, is first powered on, it draws a significantly higher current than during its steady-state operation. This surge is due to the sudden demand for energy to establish magnetic fields in inductive components. The Decay Over Time shows when the SOS decays rapidly within a short period, transitioning to the normal operating current. This decay is a critical aspect of inrush current behavior and can be modeled using various mathematical functions.

The SOS component can be modeled using different approaches. One common method used in approximated scenarios is a reciprocal function such as $\frac{1}{x}$ that might be used to describe the initial rapid drop in current.

The set of SySUPs with the added SOS, Ψ¨apϑ, is defined as:

(43) Ψ¨apϑ=ψ¨iϑi=1|Ψ¨apϑ|

where the resulting SySUP with the added SOS, ψ¨ϑ, is defined as:

(44) ψ¨ϑ=ωjλ+ξj+ϑj1+jj=1eλι−eλoλ∈Λψ^p,ψ¨ϑ∈Ψ¨apϑ

where $\vartheta_{j}$ is the SOS coefficient that follows a normal distribution function as:

(45) $$\begin{array}{c} \vartheta =N\left(\mu^{\vartheta },\sigma^{\vartheta }\right) \end{array}$$

The term $\frac{\vartheta_{j}}{1+j}$ represents a reciprocal function in which this models of the SOS component simulates the behavior of SOS current at each state.

### 8.3. The Ripple Component

In addition to the SOS component, appliance profiles may also exhibit ripple components. Ripple refers to the small, periodic oscillations in the electrical current or voltage within the steady-state periods of an appliance’s operation. It shows as temporal fluctuation in the state power value in a sinusoidal form. These ripples can arise from various factors such as switching operations of internal components, power supply noise, or inherent characteristics of the device’s operation.

The set of SySUPs with the added ripple, Ψ¨apϱ, is defined as:

(46) Ψ¨apϱ=ψ¨iϱi=1|Ψ¨apϱ|

where the resulting SySUP with the added ripple, ψ¨ϱ, is defined as:

(47) ψ¨ϱ=ωjλ+ξj+ϑj1+j+γsinjρj=1eλι−eλoλ∈Λψ^p,ψ¨ϱ∈Ψ¨apϱ

where two parameters control the behavior of the ripple: the ripple amplitude, γ, and the ripple period length, ρ. The amplitude γ determines the magnitude of the oscillations in the ripple, while the period length ρ dictates the frequency of these oscillations, or how quickly they repeat over time.

The values of these parameters are selected based on a normal distribution, which allows for realistic variability in the synthetic appliance profiles. Specifically:

(48) $$\begin{array}{c} \rho =N\left(\mu^{\rho },\sigma^{\rho }\right)\;,\;\gamma =N\left(\mu^{\gamma },\sigma^{\gamma }\right) \end{array}$$

here, $\mu^{\rho }$ and $\sigma^{\rho }$ are the mean and standard deviation of the period length, respectively, while $\mu^{\gamma }$ and $\sigma^{\gamma }$ are the mean and standard deviation of the ripple amplitude. By sampling from these normal distributions, each synthetic SUP can exhibit unique but realistically varying ripple characteristics. This stochastic approach ensures that the synthetic profiles capture the natural diversity observed in real appliance operation.

### 8.4. State Edge Position Variation

The last parameter that controls the shape of SySUPs is the Exact Edge Position (EEP), *ℓ*. This parameter introduces a variation factor to the position (sample index) of the two exact edges that define the boundaries of a state as defined in Equation (22). By varying the exact positions of these edges, the synthetic SUPs can better mimic the natural variability observed in real appliance profiles.

The set of SySUPs with the state edge position variation factor, Ψ¨apℓ, is defined as:

(49) Ψ¨apℓ=ψ¨iℓi=1|Ψ¨apℓ|

where the resulting SySUP with the added EEP, ψ¨ℓ, is defined as:

(50) ψ¨ℓ=ωjλi+ξj+ϑj1+j+γsinjρj=1(eiι+ℓiι)−(eio+ℓio)λi∈Λψ^p,ψ¨ℓ∈Ψ¨apℓλi=(eio+ℓio,eiι+ℓiι,ωλ)

such that:

(51) $$\begin{array}{c} 0\leq e_{i}^{o}+\ell_{i}^{o}<e_{i}^{\iota }+\ell_{i}^{\iota }\leq e_{i+1}^{o}+\ell_{i+1}^{o} \end{array}$$

The variation in the exact edge positions, $\ell_{i}^{o}$ and $\ell_{i}^{\iota }$, is sampled from a normal distribution. This stochastic approach ensures that the edges of the states are not fixed but exhibit natural fluctuations, thereby adding realism to the synthetic profiles. Specifically:

(52) $$\begin{array}{c} \left\{\ell_{i}^{o},\ell_{i}^{\iota },\ell_{i+1}^{o}\right\}\in N\left(\mu_{i}^{\ell },\sigma_{i}^{\ell }\right) \end{array}$$

here, $\mu_{i}^{\ell }$ and $\sigma_{i}^{\ell }$ are the mean and standard deviation of the edge position variations, respectively. By sampling from these normal distributions, each synthetic SUP can exhibit unique, yet realistically varying state boundaries. This method ensures that the synthetic profiles can accurately reflect the dynamic nature of real appliance operation, where the exact start and end times of states can vary due to numerous factors such as load conditions, user interactions, and inherent appliance behavior.

## 9. Evaluation

To evaluate the impact of the tuning parameters explained in the previous section on the SySUP with respect to the SUP, two evaluation metrics are defined: $\bar{\delta }$ and $\bar{\kappa }$. The first evaluation metric, $\bar{\delta }$, is designed to measure the average similarity between the SySUPs and the actual SUPs for a given appliance and operation mode. This metric uses the Dynamic Time Warping (DTW) distance, a well-known method for measuring similarity between time series data. The DTW distance is normalized by the sum of the lengths of the sequences being compared, ensuring that the metric is scale-invariant. The formal definition of $\bar{\delta }$ is defined as follows:

(53) δ¯Ψ¨apξ,Ψap=1|Ψ¨apξ|∑j=1|Ψ¨apξ|1|Ψap|∑i=1|Ψap|DTWψjp¨ξ,ψip|ψip|+|ψjp¨|

here, $\bar{\delta }$ represents the average DTW distance between each synthetic SUP, ψ¨apϑ∈Ψ¨apξ, and every actual SUP, $\psi_{a}^{p}\in \Psi_{a}^{p}$. The parameter *a* refers to the appliance, and *p* refers to the operation mode. The normalization factor, $|\psi_{i}^{p}\left|+\right|\ddot{\psi_{j}^{p}}|$, is the sum of the sequence lengths, ensuring that the comparison is fair across different sequence lengths.

The second evaluation metric, $\bar{\kappa }$, is designed to measure the consistency of the DTW distances between the synthetic and actual SUPs. This metric calculates the standard deviation of the DTW distances for each synthetic SUP and then averages these standard deviations. This provides insight into the variability of the synthetic SUPs relative to the actual SUPs. The formal definition of $\bar{\kappa }$ is as follows:

(54) κ¯Ψ¨apξ,Ψap=1|Ψ¨apξ|∑j=1|Ψ¨apξ|σ1|Ψap|∑i=1|Ψap|DTWψjp¨ξ,ψip|ψip|+|ψjp¨|j=1|Ψ¨apξ|

where $\bar{\kappa }$ represents the average of the standard deviations of the DTW distances between each synthetic SUP, ψ¨apξ∈Ψ¨apξ, and every actual SUP, $\psi_{a}^{p}\in \Psi_{a}^{p}$. The notation σ denotes the standard deviation. By averaging the standard deviations, $\bar{\kappa }$ provides a measure of how consistently the synthetic SUPs match the actual SUPs in terms of their DTW distances.

Together, these metrics, $\bar{\delta }$ and $\bar{\kappa }$, provide a comprehensive evaluation of the synthetic SUPs. $\bar{\delta }$ assesses the overall similarity, while $\bar{\kappa }$ evaluates the consistency of this similarity across different synthetic SUPs. This dual approach ensures a robust evaluation of the synthetic profiles against the real-world data, highlighting both the average performance and the variability of the synthetic SUPs.

### 9.1. Evaluating the Effect of the White Noise Component

**Figure 8:**
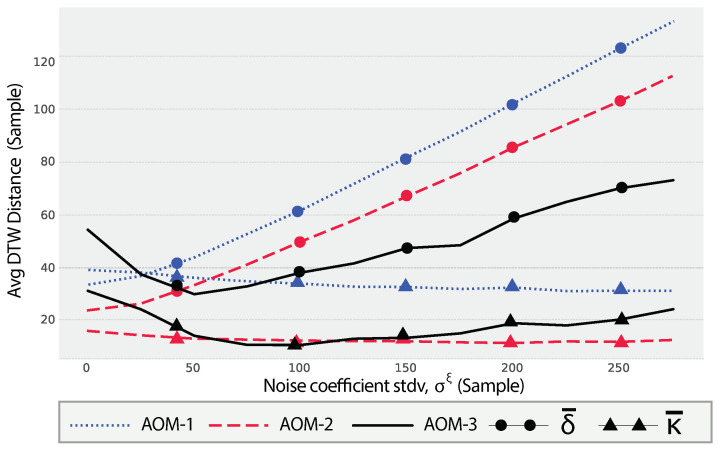
The impact of changing the noise coefficient, ξ, on the values of the distance mean, $\bar{\delta }$, for a dryer.

The provided plot in Figure 8 illustrates the impact of the noise coefficient, ξ, on the Dynamic Time Warping (DTW) distance metrics for a dryer appliance across three different Appliance Operation Modes (AOMs). The metrics $\bar{\delta }$ and $\bar{\kappa }$, as defined in Equation (53) and Equation (54), respectively, are used to assess the performance and consistency of synthetic SUPs (ψ¨apξ) relative to the real SUPs ($\Psi_{a}^{p}$).

The plot shows the effect of the noise coefficient standard deviation on the DTW distance metrics across three AOMs for a dryer. The range of $\sigma^{\xi }$ values is from 1 to 300 samples, with a distribution mean $\mu^{\xi }=0$.

For AOM-1, the average DTW distance ($\bar{\delta }$) shows a continuous increasing trend as $\sigma^{\xi }$ increases. This indicates that increasing noise levels result in higher discrepancies between the synthetic and real SUPs. The consistency metric ($\bar{\kappa }$) for AOM-1 remains relatively constant, suggesting that the variability in DTW distances does not change significantly with increasing noise levels. For AOM-2, the average DTW distance ($\bar{\delta }$) initially decreases when $1\leq \sigma^{\xi }\leq 50$, indicating that the added noise contributes to increasing the similarity between synthetic and real SUPs. However, beyond this range, $\bar{\delta }$ starts increasing, which means higher noise values lead to a decrease in similarity. The consistency metric ($\bar{\kappa }$) for AOM-2 remains low and stable, indicating consistent performance in terms of DTW distances. For AOM-3, the average DTW distance ($\bar{\delta }$) exhibits a slight initial decrease followed by a continuous increase as $\sigma^{\xi }$ increases. This pattern suggests that initially, small noise levels may help in improving the similarity between synthetic and real SUPs, but as noise levels increase, the similarity decreases. The consistency metric ($\bar{\kappa }$) for AOM-3 shows less fluctuation, indicating high consistency in DTW distances.

### 9.2. Evaluating the Effect of the Switch-On Surge Component

**Figure 9:**
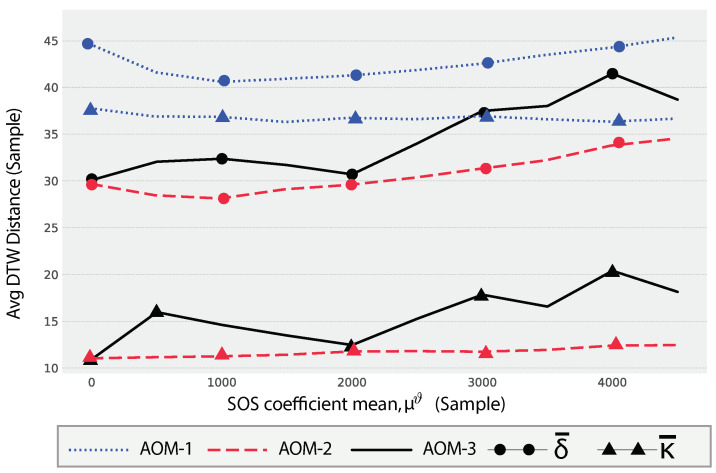
The impact of changing the SOS coefficient, ϑ, on the values of the distance mean, $\bar{\delta }$, for a dryer.

The provided plot in Figure 9 illustrates the impact of the SOS coefficient, ϑ, on the DTW distance metrics for a dryer appliance across three different AOMs. The metrics $\bar{\delta }$ and $\bar{\kappa }$, as defined in Equations (53) and (54), are used to assess the performance and consistency of synthetic SUPs (ψ¨apϑ) relative to the real SUPs ($\Psi_{a}^{p}$).

The plot shows the effect of the SOS coefficient mean on the DTW distance metrics across three AOMs for a dryer. The range of $\mu^{\vartheta }$ values is from 1 to 5000 samples, with a distribution mean $\sigma^{\vartheta }=100$. For AOM-1, the average DTW distance ($\bar{\delta }$) starts at a relatively high value followed by a slight decrease that indicates the addition of the SOS components contributes to decreasing the distance between the SUPs and SySUPs. The curve then shows a slight increasing trend as $\mu^{\vartheta }$ increases. This indicates that changes in the SOS coefficient mean lead to a gradual increase in the discrepancy between the synthetic and real SUPs. The consistency metric ($\bar{\kappa }$) for AOM-1 also follows an increasing trend, suggesting that the variability in DTW distances increases with higher values of $\mu^{\vartheta }$.

AOM-2 shows a similar behavior to AOM-1. The average DTW distance ($\bar{\delta }$) shows a stable pattern, starting at a lower value compared to AOM-1 and remaining relatively constant with slight fluctuations. The consistency metric ($\bar{\kappa }$) for AOM-2 remains low and stable, indicating a consistent performance in terms of DTW distances.

For AOM-3, the average DTW distance ($\bar{\delta }$) exhibits an initial increase followed by a decrease, and then rises again as $\mu^{\vartheta }$ increases. This non-linear pattern suggests that the SOS coefficient impacts the SUPs differently at various levels. Low and high values of SOS coefficient increase the distance difference between SUPs and SySUPs, while moderate values of $\mu^{\vartheta }\approx 2000$ samples minimizes the distance difference. The consistency metric ($\bar{\kappa }$) for AOM-3 shows some fluctuations, indicating varying levels of distance consistency across different $\mu^{\vartheta }$ values.

### 9.3. Evaluating the Effect of the Ripple Component

**Figure 10:**
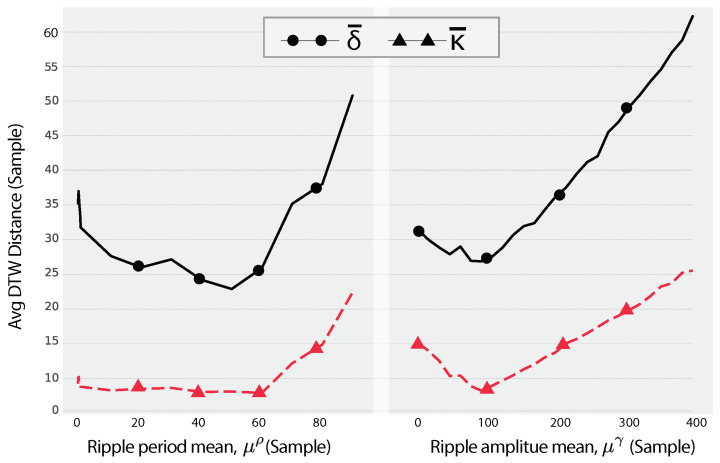
The impact of changing the ripple parameters, ρ and γ, on the values of the distance mean, $\bar{\delta }$, for a dryer.

To evaluate the impact of the ripple coefficient, the metrics in Equations (53) and (54) are plotted in Figure 10 for a single AOM for a dryer. The distance metric, $\bar{\delta }$, evaluates the impact of the ripple parameters on the SySUP, ψ¨apϱ, with respect to the SUPs, $\Psi_{a}^{p}$, while $\bar{\kappa }$ reflects the consistency of distances, $\bar{\delta }$.

The left plot in Figure 10 illustrates the effect of the ripple period mean $\mu^{\rho }$ on the DTW distance metrics. As $\mu^{\rho }$ increases, the average DTW distance ($\bar{\delta }$) initially decreases slightly, indicating an initial improvement in similarity between the synthetic and real SUPs. However, after a certain point, $\bar{\delta }$ begins to increase sharply, suggesting that excessively long ripple periods introduce significant deviations from the real SUPs. This trend reflects the non-linear impact of the ripple period on the overall shape and timing of the SUPs.

The right plot in Figure 10 shows the effect of the ripple amplitude mean $\mu^{\gamma }$ on the DTW distance metrics. Similarly to the effect of $\mu^{\rho }$, the plot initially decreases slightly, indicating an initial improvement in similarity between the synthetic and real SUPs. As $\mu^{\gamma }$ then increases, the average DTW distance ($\bar{\delta }$) consistently rises, indicating that larger ripple amplitudes lead to greater discrepancies between the synthetic and real SUPs. This increase is less sharp more gradual compared to the ripple period impact, suggesting a more predictable but significant effect of amplitude changes on SUP similarity.

For both ripple parameters, the consistency metric ($\bar{\kappa }$) shows an increasing trend, but with different patterns. In the case of $\mu^{\rho }$, $\bar{\kappa }$ remains relatively stable at lower values before increasing sharply, mirroring the trend observed in $\bar{\delta }$. This indicates that the consistency of DTW distances remains stable until the ripple period becomes excessively long. For $\mu^{\gamma }$, $\bar{\kappa }$ increases more steadily, indicating that larger ripple amplitudes consistently introduce more variability in the DTW distances.

Both ripple parameters, $\mu^{\rho }$ and $\mu^{\gamma }$, exhibit a significant impact on the DTW distance metrics, although in different ways. The ripple period mean affects the SUP similarity in a non-linear fashion, with an initial decrease followed by a sharp increase in $\bar{\delta }$, while the ripple amplitude mean shows a more straightforward increasing trend. This indicates that while both parameters are crucial for generating realistic synthetic SUPs, their effects on the DTW distance metrics differ in nature.

### 9.4. Evaluating the Effect of the State Edge Position Variation

**Figure 11:**
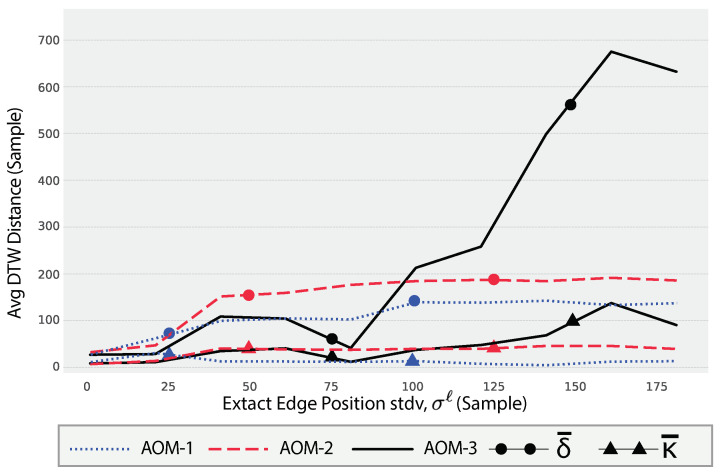
The impact of EEP factor, *ℓ*, on the values of the distance mean, $\bar{\delta }$, for a dryer.

The plot in Figure 11 depicts the impact of the EEP factor, *ℓ*, on the DTW distance metrics for a dryer appliance across three different AOMs: AOM-1, AOM-2, and AOM-3. The metrics $\bar{\delta }$ and $\bar{\kappa }$, as defined in Equations (53) and (54), respectively, are used to assess the performance and consistency of synthetic SUPs (ψ¨apℓ) relative to the real SUPs ($\Psi_{a}^{p}$).

As the standard deviation of the EEP ($\sigma^{\ell }$) increases, the average DTW distance ($\bar{\delta }$) shows a significant increasing trend, particularly for AOM-3. This indicates that larger variations in the edge positions lead to greater discrepancies between the synthetic and real SUPs. This trend is less pronounced in AOM-1 and AOM-2, suggesting that the impact of EEP variation may be more critical for certain operation modes. The different behaviors observed in AOM-1, AOM-2, and AOM-3 indicate that the effect of EEP variation is dependent on the specific operational characteristics of each mode. AOM-3 exhibits a more pronounced increase in $\bar{\delta }$ with increasing $\sigma^{\ell }$, which could be due to more complex or sensitive operational patterns that are highly affected by edge position shifts.

The consistency of the DTW distances, represented by the metric $\bar{\kappa }$, remains relatively stable across different values of $\sigma^{\ell }$. This implies that while the average distance ($\bar{\delta }$) increases, the variability of these distances does not fluctuate significantly. This stability in $\bar{\kappa }$ suggests a uniform impact of the EEP variations across different samples within each AOM.

## 10. Conclusions and Future Work

In this paper, we presented HYDROSAFE, a novel hybrid deterministic-probabilistic model for generating synthetic appliance power consumption profiles. Our approach combines data-driven analysis with stochastic elements to enhance realism and variability. The application of DTW and MDT algorithms ensures accurate clustering and profile characterization, while the probabilistic adjustments simulate realistic usage patterns. Our evaluation demonstrates that HYDROSAFE effectively replicates real-world data, offering a valuable tool for developing and testing energy management systems. The results show a high similarity between original and synthetic profiles, with an average distance of ten samples at a 1 Hz sampling rate.

Future work will explore extending HYDROSAFE to incorporate more complex appliance interactions, such as recommender systems, and expanding its application to various residential environments, thus providing a robust test bed for validating analytical algorithms and energy management solutions. Additionally, future studies will focus on validating the model by comparing its outputs with experimental data, particularly in terms of power curves and energy consumption, to assess the differences between the proposed model and the actual behavior of functioning systems.

## Figures and Tables

**Table 1:** A comparison of HYDROSAFE and publicly available synthetic datasets and simulators.

Simulator	No. Appliances	Availability	Sampling Rate	Scope	Description
Henriet et al. [46]	66	Public	0.033 Hz	Commercial	SHED is a stochastic-based comprehensive framework for energy disaggregation in commercial buildings, including a statistical analysis of differences between commercial and residential buildings, a generative model for simulating high-frequency current waveforms utilizing the Semi Non-negative Matrix Factorization (SNMF) algorithm [48].
Chen et al. [44]	25	Public	1 Hz	Residential	SmartSim is a device-accurate, NILM-TK integrated [49], smart home energy trace generator that generates complete datasets for homes with second-level energy data through a generation pipeline that utilizes historical data, Distribution learning, Event marking, and Trace Generation processes.
Buneeva et al. [42]	14	N/A	1 Hz	Residential	AMBAL is a NILM-TK integrated system for automatically generating realistic synthetic power consumption traces represented as sequences of parameterized signatures, minimizing complexity for desired accuracy.
Zhao et al. [50]	N/A	N/A	N/A	Residential	A data generation model based on Markov chains and Variational Autoencoders (VAE) to simulate diversified and random electricity consumption data for household appliances, accounting for the residential behavior and usage patterns in Chinese households.
Thorve et al. [17]	7	Public	Hourly	Residential	A large-scale digital-twin dataset of residential energy use for the contiguous United States, featuring synthetic hourly energy use profiles for the U.S. population using census data, statistical methods, activity-related attributes through regression models and survey data.
Donnal [51]	Variable	Public	Variable	Residential	NILM-Synth is a synthetic dataset generation tool that creates realistic power waveforms by superimposing extracted exemplars from live power data using existing NILM infrastructure.
Ezhilarasi et al. [40]	N/A	Public	30 min	N/A	Smart meter-SDG is a Smart Meter Synthetic Data Generator using the FBProphet library based on the UK Power Networks project.
Meiser et al. [52]	N/A	Public	N/A	Residential	SynTiSeD is a probabilistic multi-agent-based simulation tool that generates synthetic energy data based on real-world data. The model is interactive and involves Behavior Modeling, residents, and appliances into account.
Klemenjak et al. [43]	21	Public	5 Hz	Residential	SynD is a synthetic energy dataset that is generated using a custom simulation process based on power consumption patterns recorded from real household constantly on, periodical, single-pattern, and multi-pattern appliances in Austria.

N/A: Information is not available.

**Table 2:** The average of the pairwise DTW distance per house per appliance.

House	Appliance	AOM-1	AOM-2	AOM-3
1	dryer	32.704	17.23	18.74
2	dryer	77.63	203.19	208.24
1	dishwasher	31.92	8.45	-
2	dishwasher	0.76	-	-
1	washer	10.04	9.064	-
2	washer	7.65	9.29	6.24

**Table 3:** The standard deviation of pairwise DTW distance per house per appliance.

House	Appliance	AOM-1	AOM-2	AOM-3
1	dryer	44.91	5.36	17.80
2	dryer	45.22	0.0	93.15
1	dishwasher	30.11	4.91	-
2	dishwasher	0.36	-	-
1	washer	3.69	3.74	-
2	washer	2.75	3.15	1.65

## Data Availability

The data is generated using the proposed model.

## Nomenclature

H	Set of households
*h*	Single household
$A^{h}$	Set of appliances of *h*
*a*	Single appliance
$P^{a}$	Set of operation modes for *a*
*p*	Operation mode (AOM)
D	Set of days
*d*	Single day
$\Omega_{a}^{d}$	Daily power consumption sequence
$\omega_{n}$	$n^{th}$ instantaneous power sample value
$n_{*}$	Last sample index in *d*
$f_{s}$	Sampling frequency
*t*	Time
$\Psi_{a}^{d,p}$	Set of SUPs in *d*
$\psi^{p}$	Single Use Profile (SUP) activated with *p*
$\hat{\psi }$	Smoothed SUP
${\ddot{\Psi }}_{a}$	Set of synthetic SUPs for *a*
$\theta_{s}$	Length of sequence *s*
$n^{s}$	Sample index when appliance is turned on
$n^{e}$	Sample index when appliance is turned off
ϕ	Empty sequence
H	HYDROSAFE generator function
	Set of tuning parameters
$\tau^{\varepsilon }$	Stand-by power threshold
M	Moving median smoother function
*W*	Sliding window size
d(x,y)	Minkowski distance between sequences x,y
*r*	Minkowski distance order
$E(\psi ,\hat{\psi })$	L2 norm of the Minkowski pariwise distance between sequences $\psi ,\hat{\psi }$
$I^{\hat{\psi }}$	Indicator vector of $\hat{\psi }$
σ(x)	Standard deviation of sequence *x*
$W^{I}$	MDT window size
$k^{l}$	Left partition of the MDT window
$k^{r}$	Right partition of the MDT window
$\Pi^{\hat{\psi }}$	Sequence of thick edges for $\hat{\psi }$
π	Single thick edge
$n^{\iota }$	Lower bound of π
$n^{o}$	Upper bound of π
$\tau^{I}$	Thick edges threshold
$\Lambda^{\hat{\psi }}$	Sequence of states for $\hat{\psi }$
λ	Single state
*R*	Size of Λ
$e^{o}$	Left exact edge of λ
$e^{\iota }$	Right exact edge of λ
eoi⌟	Rising exact edge
eoi⌝	Falling exact edge
$d_{e}\left(q_{i},p_{j}\right)$	Euclidean distance between $q_{i},p_{j}$
DDTWSC	SUPs clustering algorithm
ϵ	Eps-neighborhood hyperparameter
μ	Minimum number of adjacent elements
$\Psi_{a}^{c}$	The core set of SUPs
$\Psi_{a}^{b}$	The border set of SUPs
$\Psi_{a}^{o}$	The outlier set of SUPs
$\Psi_{a}^{*}$	directly density-reachable SUPs
*ℵ*	DTW distance matrix
$DTW(\psi^{p_{i}},\psi^{p_{j}})$	DTW distance between $\psi^{p_{i}},\psi^{p_{j}}$
$\bar{\delta }\left(\psi^{p_{i}},\psi^{p_{j}}\right)$	Normalized pairwise DTW distance between $\psi^{p_{i}},\psi^{p_{j}}$
Δ	Normalized pairwise distance matrix
μ	Mean value
N	Normal distribution
ξ	Noise coefficient
ψ¨ξ	SySUP with added noise
$\bar{\delta }\left(\ddot{\psi },\Psi \right)$	Mean DTW distance between a SySUP and corresponding SUPs
$\bar{\kappa }\left(\ddot{\psi },\Psi \right)$	Mean of standard deviations of DTW distance between a SySUP and corresponding SUPs
ψ¨ϑ	SySUP with added SOS
ϑ	SOS coefficient
ψ¨ϱ	SySUP with added ripple
ϱ	Ripple coefficient
γ	Ripple amplitude
ρ	Ripple period length
*ℓ*	Exact Edge Position (EEP)
ψ¨ℓ	SySUP with variant EEPs
